# Comparison effects of two muscle relaxant strategies on postoperative pulmonary complications in transapical transcatheter aortic valve implantation: a propensity score-matched analysis

**DOI:** 10.1186/s13019-023-02166-9

**Published:** 2023-02-01

**Authors:** Hong Yu, Yiding Zuo, Zhao Xu, Dailiang Zhao, Jianming Yue, Lulu Liu, Yingqiang Guo, Jiapeng Huang, Xiaoqian Deng, Peng Liang

**Affiliations:** 1 Department of Anesthesiology, West China Hospital, Sichuan University, No.37 Guoxue Alley, Chengdu, 610041 China; 2 Department of Cardiovascular Surgery, West China Hospital, Sichuan University, Chengdu, 610041 China; 3 Department of Anesthesiology and Perioperative Medicine, University of Louisville, Louisville, KY USA; 4 Department of Anesthesiology, Day Surgery Center, West China Hospital, Sichuan University, No.37 Guoxue Alley, Chengdu, 610041 China

**Keywords:** Transcatheter aortic valve implantation, Neuromuscular blockade, Postoperative pulmonary complications, Rocuronium/sugammadex, Cisatracurium/neostigmine

## Abstract

**Background:**

Prior studies have reported conflicting results on the effect of sugammadex on postoperative pulmonary complications (PPCs) and research on this topic in transapical-transcatheter aortic valve implantation (TA-TAVI) was sparse. The current study aimed to investigate whether there were differences in the incidence of PPCs between two muscle relaxant strategies (rocuronium/sugammadex vs. cisatracurium/neostigmine) in patients undergoing TA-TAVI.

**Methods:**

This retrospective observational study enrolled 245 adult patients underwent TA-TAVI between October 2018 and January 2021. The patients were grouped according to the type of muscle relaxant strategies (115 with rocuronium/sugammadex in the R/S group and 130 with cisatracurium/neostigmine in the C/N group, respectively). Pre- and intraoperative variables were managed by propensity score match (PSM) at a 1:2 ratio. PPCs (i.e., respiratory infection, pleural effusion, pneumothorax, atelectasis, respiratory failure, bronchospasm and aspiration pneumonitis) were evaluated from the radiological and laboratory findings.

**Results:**

After PSM, 91 patients in the R/S group were selected and matched to 112 patients in the C/N group. Patients in the R/S group showed lower PPCs rate (45.1% vs. 61.6%, *p* = 0.019) compared to the C/N group. In addition, the R/S group showed significant shorter extubation time (7.2 ± 6.2 vs. 10.3 ± 8.2 min, *p* = 0.003) and length of hospital stay (6.9 ± 3.3 vs. 8.0 ± 4.0 days, *p* = 0.034).

**Conclusion:**

The rocuronium/sugammadex muscle relaxant strategy decreases the incidence of PPCs in patients undergoing TA-TAVI when compared to cisatracurium/neostigmine strategy.

*Trial registration* ChiCTR, ChiCTR2100044269. Registered March 14, 2021-Prospectively registered, http://www.Chictr.org.cn.

**Supplementary Information:**

The online version contains supplementary material available at 10.1186/s13019-023-02166-9.

## Introduction

Aortic valve disease is a common acquired valve diseases in adults [1], and aortic valve replacement (AVR) has been the only effective treatment which provides symptomatic relief and long-term survival [2]. Recently, transcatheter aortic valve implantation (TAVI) has gained increasing acceptance as a safe and efficient alternative for patients with severe aortic valve disease [3–5].

Because of the minimally invasive nature of the TAVI approach, patients undergoing TAVI represent a suitable cohort for early recovery [6]. However, transapical-TAVI (TA-TAVI) procedures need sufficiently profound neuromuscular blockade (NMB) during surgery which improves surgical conditions by inhibiting skeletal muscle movements. Therefore, postoperative residual NMB is one of the major hurdles for a faster recovery after surgery as it increases the risk of postoperative pulmonary complications (PPCs), such as hypoxemia [7], aspiration-induced pneumonia and reintubation [8]. Furthermore, patients receiving TAVI often having underlying illnesses such as intrinsic lung disease (e.g., chronic obstructive pulmonary disease, COPD) and pulmonary dysfunction secondary to heart failure which increase their susceptibility to PPCs [9]. Therefore, immediate restoration of patients’ muscle strength at the end of TA-TAVI procedures are warranted for fast-track anesthesia to decrease respiratory problems.

Traditionally, acetylcholinesterase (AChE) inhibitors (e.g. neostigmine,) are commonly used to reverse NMB. However, the speed of neuromuscular function recovery is unpredictable [10]. In addition, coadministration of choline antagonists is required to minimize muscarinic adverse effects (e.g. bradycardia, bronchoconstriction, hypersalivation) [11]. Sugammadex, a modified γ-cyclodextrin, was designed for the reversal of NMB by encapsulating the steroidal NMB agents such as rocuronium and vecuronium [12]. Sugammadex enables the reversal of deep NMB [13]. Furthermore, sugammadex has no major adverse effects, including adverse cardiovascular effects, due to lack of endogenous targets [14].

Despite these advantages, the effects of sugammadex on PPCs are controversial [15–21]. Especially, there are no evidence on sugammadex in high risk PPCs patients, including TA-TAVI cases. The aim of this study was to investigate the relationship between PPCs and two muscle relaxant strategies (rocuronium/sugammadex vs. cisatracurium/neostigmine) in TA-TAVI. Our primary outcome was a composite of in hospital PPCs and we hypothesized that there could be a significant reduction of PPCs in TA-TAVI patients who received rocuronium/sugammadex. We present the following article in accordance with the STROBE reporting checklist.

## Methods

Ethical approval for this retrospective observational study (No. 2019(591)) was provided by the Institutional Review Board (IRB) of West China Hospital of Sichuan University (Chairperson Prof Liu Lun-xu) on 18 December 2019. The requirement of informed consent was waived due to the retrospective nature of the analysis. The trial was registered at Chictr.org.cn (ChiCTR2100044269).

Data from adult patients who underwent TA-TAVI with endotracheal intubation and were successfully extubated in the operating room between August 2018 and January 2021 were collected retrospectively. Exclusion criteria included intubation before operating room arrival and extubation in the intensive care unit (ICU). In addition, patients with conversion to cardiopulmonary bypass (CPB) were excluded and those with incomplete or missing data were also excluded from this study.

Since August 2018, our institution implemented enhanced recovery after surgery protocols in TA-TAVI patients and patients were routinely extubated in the operating room. Anesthesia was induced with 0.1–0.2 μg/kg of sufentanil, 1 mg of midazolam, 1–2 mg/kg of propofol as necessary. Tracheal intubation was facilitated with either 0.6 mg/kg rocuronium or 0.2 mg/kg cisatracurium. Topical anesthesia of the glottis was performed by spraying with 3 ml of 2% lidocaine before intubation. Anesthesia was maintained with sevoflurane or desflurane, 1.0–1.3 minimum alveolar concentration (MAC), 0.4 μg/kg/min of dexmedetomidine, 0.1–0.3 μg/kg/min of remifentanil, and 2 mg/kg/h of lidocaine. The maintenance of effective concentrations was adjusted to achieve a target bispectral index (BIS) value of 40–60. Tropisetron 5 mg was administrated intravenously for nausea or vomiting prophylaxis. Flurbiprofen 50 mg was administrated before the end of surgery (unless contraindicated). 20 ml of 0.5% ropivacaine was used for intercostal nerve blockade before skin closure. Dexmedetomidine was discontinued 40 min before the end of the surgery. Sevoflurane (if used) was discontinued and changed to desflurane at least 30 min before the end of the procedure. At the end of surgery, remifentanil and desflurane were discontinued. Fresh gas flow of 8 L/min 100% oxygen were used to wash out inhalation anesthetics. Of note, train-of-four (TOF) ratio monitoring was not routinely used in our institution because of the limited resources, and anesthesiologists used one of two protocols for extubation. For the rocuronium/sugammadex protocol (the R/S group), a whole bottle of 200 mg sugammadex (> 2 mg/kg, the maximal body weigh was less than 100 kg) was administrated immediately after skin closure for patients who received rocuronium. For the cisatracurium/neostigmine protocol (the C/N group), neostigmine 0.04 mg/kg and atropine 0.02 mg/kg were administrated after spontaneous breathing recovery but not completely off the ventilator (insufficient tidal volume and/or frequency) for patients who received cisatracurium. For all cases, the tracheal extubation was carried out when patients were fully awake, responding to commands to open eyes, squeezing hands and lifting head for more than 5 s, and with adequate tidal volume and inspiratory force. The patient was then transferred to the ICU for further care.

Demographic and clinical data were collected from patients’ electronic medical records, including preoperative comorbidities, anesthetic records, surgery-related data and chest computed tomography reports. All medical records were collected by Dai-liang Zhao and Jian-ming Yue who were blinded to the purpose of this study, and all of the researchers were blinded to the study data until after statistical outcomes were generated.

The primary outcome was occurrence of pulmonary complications during hospitalization according to European perioperative clinical outcome (EPCO) guidelines (respiratory infection, pleural effusion, pneumothorax, atelectasis, respiratory failure, bronchospasm and aspiration pneumonitis, methods in Additional file 1: Table S1) [22]. We considered respiratory failure to be complications only when either noninvasive or invasive mechanical ventilation for oxygen therapy was initiated. All TA-TAVI patients routinely underwent their first chest computed tomography on the first or second postoperative day. Follow-up computed tomography was performed in patients with symptoms such as fever, coughing and sputum or in those with abnormalities on the first radiograph. We reviewed the radiological results until patients’ discharge from hospital.

The secondary outcomes were: (1) extubation time: defined as from completion of surgery to extubation; (2) the length of stay (LOS) in ICU and (3) LOS in the hospital: defined as from surgery completion to discharge.

### Statistical analysis

Propensity score matching was performed to minimize the risk of selection bias and confounder effects between the two groups. The patients were matched at a 1:2 ratio with a caliper of 0.2. Propensity scores were calculated with a logistic regression analysis, and based on the covariates shown in Table 1. An absolute standardized mean difference (SMD) less than 10% was considered to support the assumption of balance between the groups [23, 24]. The continuous variables were expressed as the mean with standard deviations or median with 25–75th percentiles. Categorical variables are shown as numbers (%). Comparisons between two groups were done both in original cohort and propensity-matched cohort. Student’s t-test was used for comparing continuous variables if the distribution was symmetric and Mann Whitney U test if nonsymmetric, and chi-square or Fisher’s exact test was used to compare categorical variables as appropriate. All data analyses were performed using SPSS version 25.0 software (SPSS Inc., IBM, Chicago, IL, USA) and a two-sided *p*-value of less than 0.05 was considered statistically significant.

**Table 1:** Baseline demographic and clinical characteristics for unmatched cohort and propensity-matched groups

Variables	Original cohort (n = 245)			Propensity-matched cohort (n = 203)			SMD
	R/S group (n = 115)	C/N group (n = 130)	*p* value	R/S group (n = 91)	C/N group (n = 112)	*p* value	
*Patient-related*							
Age (y)	71.7 ± 6.1	70.5 ± 6.1	0.110	71.1 ± 6.0	71.0 ± 5.9	0.883	0.042
Male gender (%)	70 (60.9)	77 (59.2)	0.794	53 (58.2)	66 (58.9)	0.921	0.022
(kg/m^2^)	23.5 ± 3.4	23.4 ± 3.5	0.812	23.4 ± 3.5	23.4 ± 3.6	0.999	0.009
Smoking (%)	34 (29.6)	32 (24.6)	0.383	22 (24.2)	29 (25.9)	0.779	0.048
NYHA functional class	4 [3–4]	3 [3–4]	0.005	4 [3–4]	3 [3–4]	0.254	0.045
EuroSCORE II (%)	10.53 ± 7.77	9.90 ± 6.36	0.487	10.08 ± 7.43	10.21 ± 6.45	0.887	0.040
LV ejection fraction (%)	55 ± 13	57 ± 12	0.278	55 ± 13	56 ± 12	0.498	0.053
> 0.5	80 (69.6)	95 (73.1)	0.574	64 (70.3)	80 (71.4)	0.918	–
0.3–0.5	32 (27.8)	31 (23.8)		25 (27.5)	28 (25.0)		–
< 0.3	3 (2.6)	4 (3.1)		2 (2.2)	4 (3.6)		–
Preop. hemoglobin	130 ± 21	132 ± 17	0.214	131 ± 20	131 ± 17	0.901	0.012
CK-MB	2.08 ± 1.26	1.91 ± 1.45	0.659	2.15 ± 1.33	1.85 ± 1.21	0.094	0.263
cTnT	27.2 ± 28.2	22.1 ± 25.9	0.146	26.1 ± 28.9	21.4 ± 19.7	0.172	0.190
BNP	2810 ± 4970	1855 ± 3035	0.067	2577 ± 5033	1869 ± 3041	0.218	0.143
Cr	94 ± 47	89 ± 43	0.368	86 ± 29	90 ± 46	0.475	0.065
GFR	70.41 ± 20.38	73.54 ± 16.94	0192	73.23 ± 17.85	72.78 ± 16.79	0.855	0.047
Aortic morbidity							
Stenosis	71 (61.7)	77 (59.2)	0.689	54 (59.3)	66 (58.9)	0.953	–
0/1/2/3/4/5/6^†^	44/7/1/4/1/56/2	53/3/0/7/8/55/0	0.525	37/3/0/4/1/44/2	46/3/0/6/7/50/0	0.569	0.075
Regurgitation	96 (83.5)	103 (79.2)	0.396	75 (82.4)	91 (81.3)	0.830	–
0/1/2/3/4/5/6^†^	19/18/6/10/19/39/4	27/11/8/11/23/48/2	0.990	16/13/6/7/17/29/3	21/11/8/8/20/43/1	0.722	0.055
Both	52 (45.2)	50 (38.5)	0.284	38 (41.8)	45 (40.2)	0.820	0.033
Preop. MV regurgitation	53 (46.1)	71 (54.6)	0.183	43 (47.2)	58 (51.8)	0.521	–
0/1/2/3/4/5/6^†^	62/27/7/11/6/1/1	59/30/20/18/1/2/0	0.219	48/24/6/8/4/1/0	54/27/16/12/1/2/0	0.478	0.049
Preop. pulmonary hypertension	8 (7.0)	7 (5.4)	0.609	6 (6.6)	7 (6.3)	0.921	–
0/1/3/4/5^†^	107/1/4/1/2	123/0/6/0/1	0.600	85/1/3/1/1	105/1/0/6/0	0.919	0.0001
Previous cardiac surgery^‡^	17 (14.8)	20 (15.4)	0.896	12 (13.2)	18 (16.1)	0.565	0.062
Comorbidities							
Atrial fibrillation	19 (16.5)	16 (12.3)	0.347	9 (9.9)	15 (13.4)	0.442	0.074
Arterial hypertension	59 (51.3)	67 (51.5)	0.971	46 (50.5)	60 (53.6)	0.668	0.044
CAD	47 (40.9)	52 (40.0)	0.890	38 (41.8)	43 (38.4)	0.626	0.067
Diabetes	18 (15.7)	11 (8.5)	0.082	10 (11.0)	11 (9.8)	0.784	0.015
Previous stroke	30 (25.6)	19 (14.6)	0.025	14 (15.4)	19 (17.0)	0.762	0.075
CRF	10 (8.7)	7 (5.4)	0.309	4 (4.4)	7 (6.3)	0.562	0.058
COPD	81 (70.4)	96 (73.8)	0.552	64 (70.3)	81 (72.3)	0.755	0.036
Pulmonary infection	11 (9.6)	6 (4.6)	0.128	5 (5.5)	6 (5.4)	0.966	0.019
PASO	61 (53.0)	69 (53.1)	0.996	45 (49.5)	56 (50.0)	0.938	0.044
*Surgery-related*							
Procedure status (urgent/emergent)	6 (5.2)	2 (1.5)	0.106	3 (3.3)	1 (0.9)	0.220	0.098
Surgical time, min	81.7 ± 23.2	82.0 ± 22.4	0.923	82.1 ± 24.2	82.3 ± 22.0	0.965	0.003
Intraoperative event							
Cardioversion	5 (4.3)	2 (1.5)	0.188	3 (3.3)	2 (1.8)	0.490	0.080
Electric defibrillation	7 (6.1)	2 (1.5)	0.059	5 (5.5)	1 (0.9)	0.054	0.206

Categorical data are expressed as numbers (%). Continuous data are expressed as mean ± SD or median (interquartile range). †. 0 = no, 1 = mild, 2 = mild to moderate, 3 = moderate, 4 = moderate to severe, 5 = severe, 6 = extremely severe; ‡. Previous cardiac surgery including pacemaker implantation, percutaneous transluminal coronary intervention and valve surgery. *BMI* Body mass index; *CAD* Coronary heart disease; *C/N* Cisatracurium/neostigmine; *COPD* Chronic obstructive pulmonary disease; *CRF* Chronic renal failure (serum creatinine level ≥ 1.5 mg/dl in men or ≥ 1.3 mg/dl in women); *EuroSCORE* European system for cardiac risk evaluation; *LV* Left ventricle; *MV* Mitral valve; *NYHA* New York Heart Association; *PASO* Peripheral arteriosclerosis obliterations; *R/S* Rocuronium/sugammadex; *SMD* Standardized mean difference

## Results

### Patient characteristics

In the initial cohort of 278 patients who underwent TA-TAVI between August 2018 and January 2021 at West China Hospital, we excluded 28 patients extubated in the ICU, 2 intubated already before operating room arrival and 3 converted to CPB. Finally, 245 patients were included in the analysis. Among them, 115 patients received rocuronium/sugammadex, and 130 patients received cisatracurium/neostigmine. Because these patients were not randomly assigned, there were statistically significant differences in NYHA functional class (*p* = 0.005) and previous stroke rate (*p* = 0.025) between the two groups.

**Fig. 1:**
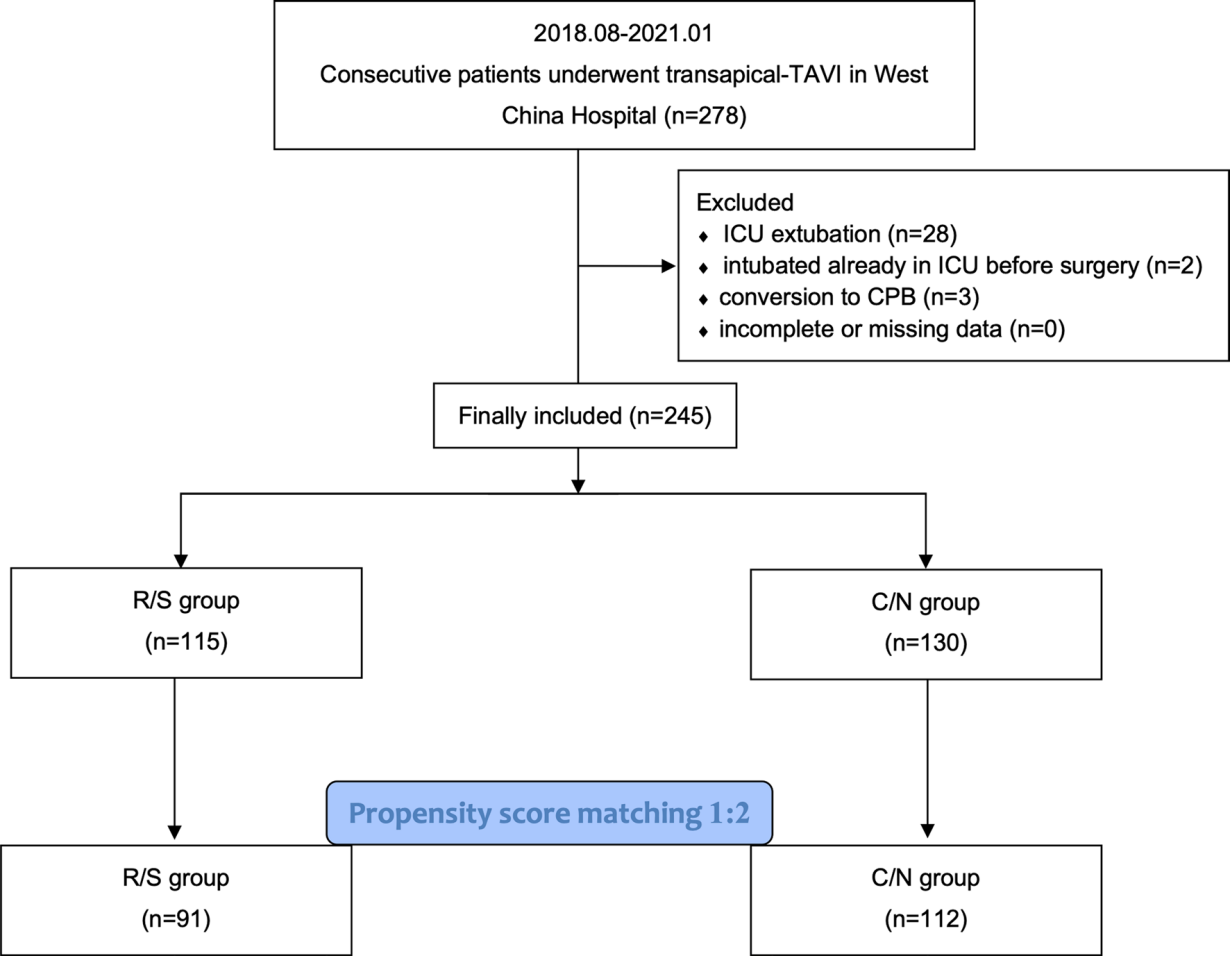
Flowchart displaying the identification of the matched pair groups. *C/N* Cisatracurium/neostigmine; *CPB* Cardiopulmonary bypass; *ICU* Intensive care unit; *R/S* Rocuronium/sugammadex; *TAVI* Transcatheter aortic valve implantation

After propensity score matching, a series of 91 patients receiving rocuronium/sugammadex matched to 112 patients receiving cisatracurium/neostigmine (Fig. 1). The patients’ characteristics and SMD values for the matched cohort are shown in Table 1. As expected, following matching, no significant difference between the two groups were detected regarding patient-related and surgery-related variables (Table 1).

### Primary outcomes

After propensity score matching, there was statistically significant differences in the PPCs rate: 45.1% in the R/S group vs. 61.6% in the C/N group (*p* = 0.028). There were no significant differences in the individual components of the primary outcome, including respiratory infection (11.0% vs. 19.6%, *p* = 0.092), atelectasis (2.2% vs. 4.5%, *p* = 0.379), pneumothorax (6.6% vs. 7.1%, *p* = 878) and respiratory failure (26.4% vs. 32.1%, *p* = 0.370). But there was statistically significant difference in the pleural effusion rate between the two groups (11.0% in the R/S group vs. 19.6% in the C/N group, *p* = 0.003). No aspiration pneumonitis nor bronchospasm occurred in either R/S or C/N group (Table 2).

**Table 2:** Postoperative pulmonary complication rate and secondary outcomes in the original cohort and propensity-matched cohort

Variables	Original cohort (n = 245)				Propensity-matched cohort (n = 203)			
	R/S group (n = 115)	C/N group (n = 130)	Estimated difference (95% CI)	*p* value	R/S group (n = 91)	C/N group (n = 112)	Estimated difference (95% CI)	*p* value
Pulmonary complications	54 (47.0)	81 (62.3)	− 15.3 (− 27.7 to − 3.0)	0.016	41 (45.1)	69 (61.6)	− 16.5 (− 30.1 to − 2.9)	0.019
Respiratory infection	10 (8.7)	23 (17.7)	− 9.0 (− 17.3 to − 0.3)	0.04	10 (11.0)	22 (19.6)	− 8.6 (− 18.4 to 1.2)	0.092
Pleural effusion	18 (15.7)	43 (33.1)	− 17.4 (− 27.9 to − 6.9)	0.002	13 (14.3)	36 (32.1)	− 17.8 (− 28.1 to − 7.5)	0.003
Atelectasis	2 (1.7)	6 (4.6)	− 2.9 (− 7.2 to 1.4)	0.206	2 (2.2)	5 (4.5)	− 2.3 (− 6.8 to 2.2)	0.379
Pneumothorax	9 (7.8)	9 (6.9)	0.9 (− 5.7 to 7.5)	0.787	6 (6.6)	8 (7.1)	− 0.5 (− 6.8 to 5.8)	0.878
Respiratory failure	33 (28.7)	43 (33.1)	− 4.4 (− 16.0 to 7.2)		24 (26.4)	36 (32.1)	− 5.7 (− 17.1 to 5.7)	0.370
Noninvasive ventilation	33 (28.7)	42 (32.3)	− 3.5 (− 15.0 to 8.0)	0.540	24 (26.4)	35 (31.3)	− 4.9 (− 16.2 to 6.4)	0.447
Reintubation	1 (0.9)	5 (3.8)	− 2.9 (− 6.6 to 0.8)	0.132	1 (1.1)	5 (4.5)	− 3.4 (− 7.4 to 0.6)	0.159
Aspiration pneumonitis	0	0	n/a	n/a	0	0	n/a	n/a
Bronchospasm	0	0	n/a	n/a	0	0	n/a	n/a
Extubation time (min)	7.1 ± 6.2	10.1 ± 8.0	− 3.0 (− 4.8 to − 1.2)	0.001	7.2 ± 6.2	10.3 ± 8.2	− 3.1 (− 5.1 to − 1.1)	0.003
LOS								
ICU, h	25.8 ± 19.8	25.0 ± 16.3	0.8 (− 3.8 to 5.4)	0.734	24.5 ± 17.1	25.2 ± 17.3	− 0.7 (− 5.5 to 4.1)	0.748
Surgery completion to discharge, d	6.8 ± 3.0	7.9 ± 4.1	− 1.1 (− 2.0 to − 0.2)	0.025	6.9 ± 3.3	8.0 ± 4.0	− 1.1 (− 2.1 to − 0.1)	0.034

Categorical data are expressed as numbers (%). Continuous data are expressed as mean ± SD. *C/N* Cisatracurium/neostigmine; *LOS* Length of stay; *ICU* Intensive care unit; *R/S* Rocuronium/sugammadex

### Secondary outcomes

After propensity score matching, the R/S group showed significant reductions in the extubation time (7.2 ± 6.2 vs. 10.3 ± 8.2 min, *p* = 0.003) and length of hospital stay (6.9 ± 3.3 vs. 8.0 ± 4.0 d, *p* = 0.034) when compared to the C/N group. However, the length of ICU stay (24.5 ± 17.1 vs. 25.2 ± 17.3 days, *p* = 0.748) were similar between two groups (Table 2).

## Discussion

This retrospective observational propensity score-matched study showed that the use of rocuronium/sugammadex compared to cisatracurium/neostigmine decreased the incidence of PPCs and pleural effusion in patients undergoing TA-TAVI. However, the incidence of respiratory infection, pneumothorax, atelectasis, respiratory failure, bronchospasm and aspiration pneumonitis did not differ significantly between the two groups. Of the secondary outcomes, the use of rocuronium/sugammadex was associated with shorter extubation time and length of hospital stay when compared to the use of cisatracurium/neostigmine.

TAVI is an efficient treatment for high-risk and intermediate risk surgical candidates with aortic valve disease, as well as those deemed to high risk to undergo open surgery [25, 26]. Among current implanted prostheses available, only J-valve system is suitable for both severe aortic valve stenosis and regurgitation patients [27–29]. However, the J-valve is still introduced through TA access which needs sufficient NMB to facilitate the surgical procedure. As a result, the use of nondepolarizing muscle relaxants could increase the risk of postoperative residual NMB and PPCs. Previous meta-analyses have shown that sugammadex reversed NMB more rapidly than neostigmine and was associated with fewer residual NMB rate (TOF ratio of less than 0.9) [30, 31]. However, prior observational [16, 18, 32] and randomized trials [15, 17, 33] have reported conflicting results on the effect of sugammadex on PPCs.

Our study showed that rocuronium/sugammadex reduced the composite PPCs rate and pleural effusion rate. Unlike other relevant studies including non-cardiac patients [15, 17, 34], the most common pulmonary complication observed in our study was pleural effusion rather than atelectasis. We considered all pleural effusion to be complications regardless the sides. Because unlike open heart surgery, the delivery catheter and implanted prostheses were inserted through the cardiac apex area on fifth intercostal space without opening the pleural cavity. Pleural effusion is a common complication after cardiac surgery as these TAVI patients are often complicated with heart failure, atrial fibrillation, peripheral vascular disease, receiving therapy with an anticoagulant or antiarrhythmic agent [35]. From this, we enrolled the preoperative cardiac function parameters (i.e., NYHA functional class, LV ejection fraction, BNP, CK-MB, cTnT, intraoperative cardioversion or electric defibrillation event) in propensity score matching. After matching, the difference of pleural effusion between the two groups remained statistically significant. In addition, the residual NMB could also contribute to pleural effusion from incomplete recovery of respiratory muscular function [36]. Sugammadex can quickly and efficiently re-establish normal muscle strength and cause less postoperative pleural effusion rate after TA-TAVI procedure. This finding was reinforced from a retrospective observational study by Han et al. [17]. They found that the postoperative pleural effusion rate was lower in patients receiving sugammadex when compared to patients receiving neostigmine, although they failed to found a significant difference of the incidence of PPCs between the groups [17]. Furthermore, previous study showed that neostigmine can adversely affect neuromuscular function and impair muscle function (genioglossus muscle and diaphragm) which was associated with respiratory complications [37, 38].

There was a higher rate of NIV in our study (29.06%) compared to other studies for non-cardiac surgeries (1.59–12.16%) [18–21]. The high rate of COPD in our study may contribute to the higher NIV use. Some studies showed sugammadex was associated with less post-extubation desaturation and consequent NIV use [18, 20]. However, in our study, the incidences of NIV (26.4% vs. 31.1%) were similar between the two groups.

In our study, the extubation time was 7.2 ± 6.2 min in the R/S group and 10.3 ± 8.2 min in the C/N group. Our study confirmed that rocuronium/sugammadex was superior to cisatracurium/neostigmine in reducing the extubation time. Lower residual NMB rate following the use of sugammadex [15, 30, 31, 33, 39] may explain the faster extubation in the rocuronium/sugammadex group. Alternatively, this difference in extubation time could be explained by the fact that in the rocuronium/sugammadex protocol reversal agents were administered upon skin closure while the cisatracurium/neostigmine protocol required return of spontaneous breathing prior to dosing of reversal agents. Our finding was consistent with reports of two randomized studies including thoracic surgery with single lung ventilation [33, 40].

Another finding of this trial was that the LOS in hospital was 1.1 days shorter in the R/S group than the C/N group. This was consistent with reports of the association between the PPCs and prolonged hospital LOS [41, 42]. However, several previous studies have not detected a reduction of hospital LOS with the use of sugammadex [15, 17, 19, 21, 34, 39, 43, 44]. It might be explained by different study population between studies. We included patients with aortic valve disease who had poor clinical conditions from older age, more comorbidities and higher European system for cardiac risk evaluation (EuroSCORE) score when compared with other studies which included non-cardiac surgery patients [15, 17, 19, 21, 34, 39, 43, 44]. As a result, the postoperative hospital LOS (7.5 days) in our study was longer than other studies (3.5–7.5 days) [15, 21, 34, 43–45] except one study including major abdominal surgery patients (12.5 days) [19].

Recapitulating the results of several studies, our study failed to detect a reduction in respiratory infection with the use of rocuronium/sugammadex [15–17, 19, 34, 40, 45]. However, the R/S group showed a significantly lower respiratory infection rate before matching (8.7% vs 17.7%, *p* = 0.04). Although there was no statistical significance after matching, the R/S group showed a relative 44% decrease of respiratory infection rate (11.0% vs 19.6%, *p* = 0.092) which was clinically significant. Actually, relevant studies involving non-cardiac surgeries reported an extremely low respiratory infection rate which ranged from 0.4 to 3.33% [15–17, 19, 34, 40, 45]. We supposed that the results in the current and relevant studies might be explained by the insufficient power of the relatively low sample size to detect the difference in respiratory infection with lower event rates. Actually, in a large sample-sized observational study which included 45,712 patients, a 47% reduced risk for respiratory infection (adjusted odds ratio, 0.53; 95% CI 0.44–0.62) was found in the sugammadex group compared to the neostigmine group [16].

This study has some limitations. First, this was a retrospective single center series of TA-TAVI. However, we used PSM based on almost all possible variables to control confounding factors. The second weakness is the lack of neuromuscular monitoring. Reversal with sugammadex in the absence of monitoring did not preclude residual neuromuscular block [46].

In conclusion, this propensity score match-based study showed that rocuronium/sugammadex decreased the incidence of PPCs in patients undergoing TA-TAVI. A sufficiently powered, prospective randomized study is warranted to confirm this effect size.

## Supplementary Information

**Additional file 1: Table S1.** The criteria for postoperative pulmonary complications.

## Data Availability

The datasets used and/or analyzed during the current study are available from the corresponding author on reasonable request.

## Abbreviations

AChE: Acetylcholinesterase
AVR: Aortic valve replacement
BIS: Bispectral index
BMI: Body mass index
CAD: Coronary heart disease
C/N: Cisatracurium/neostigmine
COPD: Chronic obstructive pulmonary disease
CPB: Cardiopulmonary bypass
CRF: Chronic renal failure
EPCO: European perioperative clinical outcome
EuroSCORE: European system for cardiac risk evaluation
ICU: Intensive care unit
LOS: Length of stay
IRB: Institutional review board
LV: Left ventricle
MAC: Minimum alveolar concentration
MV: Mitral valve
NIV: Noninvasive ventilation
NMB: Neuromuscular blockade
NYHA: New York Heart Association
PASO: Peripheral arteriosclerosis obliterations
PPCs: Postoperative pulmonary complications
PSM: Propensity score match
R/S: Rocuronium/sugammadex
SMD: Standardized mean difference
TA-TAVI: Transapical-transcatheter aortic valve implantation
TOF: Train-of-four
