# Supplementary material for: Comparison effects of two muscle relaxant strategies on postoperative pulmonary complications in transapical transcatheter aortic valve implantation: a propensity score-matched analysis
Source: J Cardiothorac Surg. 2023 Feb 1;18:50. doi: 10.1186/s13019-023-02166-9 (PMC9890810; doi:10.1186/s13019-023-02166-9)
Supplement: Supplementary file 1 — Additional file 1: Table S1. The criteria for postoperative pulmonary complications. [file 13019_2023_2166_MOESM1_ESM.doc]

**Supplemental Table S1. The criteria for postoperative pulmonary complications**

| Complication | Definition |
| --- | --- |
| Respiratory infection | Patient has received antibiotics for a suspected respiratory infection and met one or more of the following criteria: new or changed sputum, new or changed lung opacities, fever, white blood cell count > 12 × 10^9^ L^-1^ |
| Pleural effusion | Chest radiograph demonstrating blunting of the costophrenic angle, loss of sharp silhouette of the ipsilateral hemidiaphragm in upright position, evidence of displacement of adjacent anatomical structures or (in supine position) a hazy opacity in one hemithorax with preserved vascular shadows |
| Atelectasis | Lung opacification with a shift of the mediastinum, hilum or hemidiaphragm toward the affected area, and compensatory over-inflation in the adjacent non-atelectatic lung |
| Pneumothorax | Air in the pleural space with no vascular bed surrounding the visceral pleura |
| Respiratory failure | Postoperative initiation of either noninvasive or invasive mechanical ventilation for oxygen therapy |
| Bronchospasm | Newly detected expiratory wheezing treated with bronchodilators |
| Aspiration pneumonitis | Acute lung injury after the inhalation of regurgitated gastric contents |
